# Longitudinal associations between prepubertal childhood total energy and macronutrient intakes and subsequent puberty timing in UK boys and girls

**DOI:** 10.1007/s00394-021-02629-6

**Published:** 2021-07-07

**Authors:** Tuck Seng Cheng, Stephen J. Sharp, Soren Brage, Pauline M. Emmett, Nita G. Forouhi, Ken K. Ong

**Affiliations:** 1 MRC Epidemiology Unit, Institute of Metabolic Science, School of Clinical Medicine, University of Cambridge, Cambridge Biomedical Campus Box 285, Cambridge, CB2 0QQ UK; 2 Bristol Medical School, Centre for Academic Child Health, University of Bristol, Bristol, UK; 3 Department of Paediatrics, University of Cambridge, Cambridge, UK

**Keywords:** ALSPAC, Dietary intake, Puberty timing, Protein, Prospective study, Random intercepts model

## Abstract

**Purpose:**

Early puberty is associated with adverse health outcomes. To identify potential modifiable factors for puberty timing, we examined the associations of prepubertal childhood macronutrient intakes with puberty timing in boys and girls.

**Methods:**

In the Avon Longitudinal Study of Parents and Children, macronutrient intakes at age 6 years were predicted using random intercepts linear regression models of dietary data at 3, 4, 7 (assessed by food frequency questionnaires) and 7.5 years (by 3-day food diaries). Timings of puberty onset (Tanner stage 2 genital or breast (B2) development) and puberty completion (voice breaking (VB) or menarche) were calculated from annual parental and child reports at 8–17 years. Age at peak height velocity (PHV) was derived from repeated height measurements at 5–20 years. Linear regression models were fit to estimate the associations of total energy (TEI) and macronutrient intakes (carbohydrate, fat, protein) with puberty timing traits, adjusting for maternal and infant characteristics.

**Results:**

Among 3811 boys, higher TEI, but no macronutrient, was associated with earlier VB. Among 3919 girls, higher TEI was associated with earlier ages at B2, PHV, and menarche. Higher protein intake but not carbohydrate or fat intake (in energy partition models) and substitution of dietary protein for carbohydrate (in nutrient density and residual models) was associated with earlier B2, PHV, and menarche in girls. Findings were not attenuated on additional adjustment for body fat percentage during adolescence.

**Conclusions:**

These findings suggest habitual total energy intakes in children, and protein intakes in girls, as potential modifiable determinants of puberty timing.

## Introduction

Puberty is the transition from childhood to adult body appearance and attainment of reproductive capacity, a process that takes 2–3 years. The timing of puberty is typically indicated in boys by age at onset of genital development or voice breaking and in girls by the age at onset of breast development or menarche, and these timings have become earlier over the last decades worldwide [1–4]. Such trends are concerning, because earlier puberty timing has been associated with adverse mental [5] and physical health outcomes [6, 7]. A recent systematic review estimated that a significant proportion of type 2 diabetes may be attributed to early menarche timing among white British women [8]. These findings highlight the need to identify potentially modifiable factors that determine puberty timing. It is well recognised that girls who experience rapid childhood growth and have overweight or obesity tend to progress through puberty earlier than their peers [9, 10]. Such findings raise the possibility that dietary intakes, whether or not acting through child growth and obesity, may be potential modifiable determinants of puberty timing [11].

Previous observational studies have examined the associations between total energy or macronutrient (i.e., carbohydrate, fat, and protein) intakes in childhood with subsequent puberty timing, but the findings are conflicting and limited mainly to girls [11]. A few studies have inconsistently reported that higher total energy intake was associated with earlier [12, 13] or later age at menarche [14, 15], whereas others reported no association between total energy intake and age at menarche [16–18] or breast development [19]. Similarly, studies have variably reported that higher total protein intake was associated with earlier [13] or later age at menarche [20]. Other studies reported no associations for protein intake with age at menarche [14–19, 21, 22], but differential associations with other puberty timing traits including later onset of breast development [19, 20], and earlier voice breaking and peak height velocity in boys and girls [22]. Negative [23], positive [24], or null associations [13–17, 19, 21] have also been reported between total fat intake and age at menarche. Higher carbohydrate intake was associated with later age at menarche in one study [21], but most studies reported no association between carbohydrate intake and puberty timing [13–15, 17–19]. Notably, those previous studies mostly relied on a single self-report of puberty timing, usually age at menarche. It is important to consider other puberty timing traits which may represent different underlying sex hormone pathways [25]. Moreover, most studies assessed dietary intake only at a single time-point and did not model the effects of isocaloric macronutrient substitutions.

Here, we aimed to examine the prospective associations of habitual energy and macronutrient intakes during prepubertal childhood with several puberty timing traits in boys and girls. We hypothesised that higher total energy intake would be associated with earlier puberty timing, predominantly in girls, given the more consistent negative association between body mass index and puberty timing observed in girls than boys [9, 10]. Since the reported associations between macronutrient intakes and puberty timing are inconsistent, we explored associations with all three main macronutrient intakes, accounting for isocaloric macronutrient substitutions. Furthermore, we explored whether any observed association was independent of adiposity during puberty.

## Methods

### Study population

The Avon Longitudinal Study of Parents and Children (ALSPAC) is an ongoing population-based birth cohort study that initially enrolled 14,541 pregnant mothers from the former Avon country of southwest England, with expected dates of delivery between 1 April 1991 and 31 December 1992 [26, 27]. The cohort included in total 14,901 children, comprising 13,988 children from those pregnancies who were alive at age 1 year and 913 children additionally recruited from age 7 years onwards. The participants were followed up at regular intervals using questionnaire- and clinic-based assessments. Further information about the study including details of all the data available are at: http://www.bristol.ac.uk/alspac/researchers/our-data/. Ethical approval for this cohort study was obtained from the ALSPAC Ethics and Law Committee and the Local Research Ethics Committees (http://www.bristol.ac.uk/alspac/researchers/research-ethics/).

Based on the same ALSPAC cohort, a similar analysis of the present question has been previously reported, but that approach was restricted to dietary intakes at 3, 7, and 10 years (considered separately) and a single outcome only in girls of dichotomous menarche status at age 12.89 ± 0.23 years [13].

### Dietary assessments

Children’s dietary intakes were reported by main carers using food frequency questionnaire (FFQ) at ages ~ 3 (mean ± SD: 3.22 ± 0.10 years) (*n* = 9797, 70.0%), ~ 4 (4.54 ± 0.10 years) (*n* = 9484, 67.8%) and ~ 7 years (6.79 ± 0.11 years) (*n* = 8234, 58.9%), and by 3-day food diaries at age ~ 7.5 years (7.5 ± 0.17 years) (*n* = 8293, 59.3%), as described in detail elsewhere [28, 29]. Further information on the plausible reporters can be found in Appendix 1.

In brief, at each age, FFQ was adapted to cover age-appropriate child’s diets and validated against 3-day food diaries administered to a 10% subcohort [13]. The FFQ items asked participants to indicate their child’s current frequency of intakes of ~ 60 food/drink groups, each with 5 response options ranging from ‘*never or rarely*’ to ‘*more than once a day*’ [28, 29]. A further 5 food/drink groups, normally consumed daily in a variety of types (bread, spreading and frying fat, milk, tea, and coffee), were covered in more detail. Nutrient intakes (per day) were estimated by multiplying the reported frequency of each food consumed by the nutrient content of a standard portion tailored to the age of the child (from the 5th edition of McCance and Widdowson’s The Composition of Foods and its supplements [30]) [28, 29]. At each age, dietary data for 223 to 329 individuals were excluded, because they had highly implausible values for total energy intakes on visual inspection of histograms (age 3: ≤ 349 kcal and ≥ 2617 kcal; age 4: ≤ 514 kcal and ≥ 3263 kcal; age 7: ≤ 545 kcal and ≥ 3970 kcal) [29].

The 3-day food diaries were posted to families prior to attending research clinics, to record all foods and drinks consumed by the child in household measures over 3 (consecutive or non-consecutive) days including 1 weekend day and 2 weekdays. At 7.5 years, majority reported 3 days of dietary intakes (*n* = 6020, 82.7%), followed by 2 days (*n* = 994, 13.7%) and 1 day (*n* = 263, 3.6%). Energy and nutrient contents of each food consumed were estimated using DIDO software [31], based on a database comprising the 5th edition of McCance & Widdowson’s Food Composition tables, as well as information from manufacturers and the National Diet and Nutrition Survey [28, 29].

### Childhood macronutrient intakes

Intake values between ages 3–7.5 years were combined and summarised using random intercepts linear regression models for total energy intake (TEI) and each macronutrient (carbohydrate, fat, and protein) [32]. In each model, age was centred to 6 years and the individual intake of each macronutrient at age 6 years was predicted by adding a constant value (i.e., the population mean intake at age 6 years) to the Best Unbiased Linear Predictor estimates (i.e., the difference between the person-specific intercept and the overall intercept).

### Assessment of pubertal development

Repeated data on children’s pubertal development were collected by an annually administered questionnaire (an adapted version of the Pubertal Development Scale) [33], which was completed by caregivers (mainly parent) from ages 8 to 13 years and by children themselves from ages 14 to 17 years (available for 4370–7017, 29.3–47.1%). Measures of puberty included genital development in boys and breast development in girls, and pubic hair in both sexes, which were reported from a choice of five Tanner stages ranging from prepuberty (stage 1) to postpuberty (stage 5). Voice breaking status in boys was reported as: ‘no’, ‘partial’ or ‘total’. Menarche in girls was reported as the date (month and year) and/or age (in years) at first occurrence of menstruation. Axillary hair in both sexes was reported as: ‘no’ or ‘yes’.

### Calculation of puberty timing traits

Repeated measures of pubertal development were synthesised to determine individual estimates of: (1) age at pubertal onset, based on Tanner stage 2 genital or breast development in boys and girls, respectively, and (2) age at pubertal completion, based on voice breaking or menarche, respectively. We similarly calculated other puberty timing traits and considered these as secondary outcomes: age at pubic hair, axillary hair, and pubertal duration (i.e., time from puberty onset to completion).

Appendix 2 describes calculations of age at onset of genital (G2), breast (B2), pubic hair (PH2), axillary hair (AH), and voice breaking (VB). There were frequent missing data on timepoints or inconsistent reports and we imputed values where possible; however, we could not calculate timings of G2, B2, PH2, and VB for 20.7%–31.7% of included individuals.

We calculated age at menarche as the earliest reported age (from reported date or age). Values for age at VB (*n* = 27) or menarche (*n* = 39) were excluded if these occurred earlier than age at G2 and B2, respectively. We calculated pubertal duration as the difference between ages at VB and G2 in boys, and between ages at menarche and B2 in girls.

We also included an objective measure of puberty timing, namely age at peak height velocity (PHV), which was derived from 46,246 measurements of standing height obtained by trained fieldworkers at clinics (Leicester height measure at 5–6 years, Harpenden Stadiometer at 7–20 years) in 5707 participants between ages 5 and 20 years [34]. The computation of PHV was performed separately in boys (*n* = 2688) and girls (*n* = 3019), by transformation of the random age intercept that indicates the timing of the height growth spurt using SuperImposition by Translation and Rotation analysis [34].

### Potential confounders

We selected potential confounders as those which have been associated with child’s dietary intakes and puberty timing [35]. These included maternal characteristics (age at delivery, passive and active smoking during pregnancy, age at menarche, education, pre-pregnancy body mass index, and parity), highest maternal or mothers’ partner’s occupational socioeconomic group at 18 weeks of gestation, and infant characteristics (birth weight, gestational age, and breastfeeding duration).

### Adiposity during adolescence

Repeat measures of body fat percentage (%BF) by dual-energy X-ray absorptiometry (Lunar prodigy) were collected biennially from age 9 to 15 years (Pearson’s correlation coefficients = 0.90–0.95). We combined these values using random intercepts linear regression to predict %BF at age 11 years in each individual, as an estimate of adiposity during adolescence. At age 11 years, about three-quarters (*n* = 4378, 75.2%) had started puberty, but most (*n* = 6025, 93.7%) had not completed puberty.

### Statistical analysis

We compared differences between excluded and included children using chi-squared test for categorical variables and t test for continuous variables.

To examine the associations of TEI and macronutrient intakes with puberty timing, we fitted multivariable linear regression models, separately in boys and girls, with adjustment for potential confounders. We fitted further models with additional adjustment for %BF at age 11 years as a potential mediator.

To explore the relative roles of macronutrients in puberty timing, we first used the *energy partition model*, which includes the energy contributions from carbohydrate, fat, and protein in the same model [36]. Then, in a more robust approach, we tested the substitution of the macronutrient of interest for another macronutrient in an isocaloric diet using the *nutrient density method* which includes percentages of energy contributed by the two macronutrients and TEI [36]. For example, in a model that includes percentages of energy from fat and protein and TEI, the resulting beta value for protein indicates the effect of 10% higher protein intake as carbohydrate (the only excluded macronutrient) intake is concurrently lower by 10%, while fat and total energy intakes are held constant (i.e., ‘substitution of protein for carbohydrate’). We also performed sensitivity analyses to examine the associations of isocaloric macronutrient substitution with puberty timing using the *residual method* which includes the residuals from the regression of each macronutrient intake on TEI [36] and thus accounts for potential underreporting of dietary intakes [37].

To inform public health implications, we conducted a series of post hoc analyses for primary puberty timing traits. We used multivariable logistic regressions to evaluate the role of intakes above or below the United Kingdom (UK) recommended values on dichotomous puberty timing status (earlier and equal to/later than the population mean by sex), using the reported Estimated Average Requirement (EAR) (1577 kcal for boys and 1483 kcal for girls at 6 years old [38]) and dietary recommended value for protein (19.7 g per day for boys and girls at age 6 years [39]). Separately, to explore potential associations between dietary intakes and nonlinear puberty timing outcomes, we categorised puberty timing traits into tertiles and used multivariable logistic regressions to estimate effects of intakes on 1st compared to 2nd tertile as reference, and 2nd compared to 3rd tertile as reference, and compared these odds ratios using Wald tests. Furthermore, we examined the associations of protein quality or type, particularly substitutions of protein from animal sources, i.e., red meat (both processed and unprocessed), white meat (poultry and fish), and dairy and eggs for protein from plant-based sources with puberty timing.

To explore the robustness of the results to missing data on dietary intake (*n* = 204, 2.7%) and more missing data on covariates (*n* = 17–1238, 0.21–16.0%; *n* = 3401, 44.0% with at least one missing), we used multiple imputation by chained equations with 50 imputed datasets [40].

All statistical analyses were performed using Stata 15.1 (StataCorp. 2017. Stata Statistical Software: Release 15. College Station, TX: StataCorp LLC).

## Results

### Participants’ characteristics

The initial sample size in the present study was 10,789 children, who were white (when mother and her partner reported they were white), full-term birth (gestational age ≥ 36 weeks), and singleton, and had mother’s age at delivery ≥ 18 years. Of these children, 7730 (71.6%) (3811 boys and 3919 girls) had data on at least one puberty timing trait and were included in the final analytical sample. The average follow-up period was 11.5 ± 2.9 years. Compared to the excluded children (*n* = 3059), mothers of those included children were more likely to be older, nulliparous, from higher socioeconomic groups, have higher education level, and less likely to smoke or have second-hand smoke exposure during pregnancy (Supplementary Table 1). Included children were more likely to be girls, were breastfed for longer, and tended to have lower intakes of total energy, carbohydrate, and fat at age 3–7 years than children who were excluded due to missing data on puberty timing.

Table 1 summarises macronutrient intakes at age 6 years, puberty timing traits, and %BF at age 11 years in boys and girls. Compared to boys, girls consumed lower total energy, relatively less energy from carbohydrate, and relatively more energy from protein, particularly from red and white meats but less from plant sources. Total energy and macronutrient intakes predicted at age 6 years were moderately-to-highly correlated with the respective intakes reported at other ages (Pearson’s correlation coefficients = 0.55–0.81) (Supplementary Table 2). Most pubertal traits occurred earlier in girls than boys, except for pubertal onset, where surprisingly G2 was reported earlier than B2 (Table 1). Correlations between pubertal traits were stronger in girls than in boys (Supplementary Table 3). Also, girls had higher %BF at 11 years than boys (Table 1).

**Table 1:** Macronutrient intakes at age 6 years, puberty timing, and body fat percentage at age 11 years in boys and girls

	Boys (*n* = 3811) mean ± SD	Girls (*n* = 3919)mean ± SD	*p*
Total energy intake, kcal	1654 ± 160	1579 ± 148	< 0.001
Total carbohydrate intake, g	207.2 ± 20.8	196.9 ± 19.4	< 0.001
Total fat intake, g	67.4 ± 7.4	64.4 ± 6.8	< 0.001
Total protein intake, g	56.6 ± 5.8	54.7 ± 5.3	< 0.001
From red meat	7.2 ± 1.8	6.9 ± 1.7	< 0.001
From white meat	9.6 ± 1.9	9.8 ± 1.9	< 0.001
From dairy and eggs	17.0 ± 3.2	16.3 ± 2.9	< 0.001
From plant sources	22.8 ± 2.6	21.6 ± 2.5	< 0.001
Percentage of energy from carbohydrate, %	50.1 ± 1.7	49.9 ± 1.5	< 0.001
Percentage of energy from fat, %	36.5 ± 1.6	36.6 ± 1.4	0.055
Percentage of energy from protein, %	13.8 ± 1.0	14.0 ± 0.9	< 0.001
From red meat	1.7 ± 0.5	1.8 ± 0.4	0.035
From white meat	2.4 ± 0.5	2.5 ± 0.5	< 0.001
From dairy and eggs	4.2 ± 0.7	4.2 ± 0.7	0.256
From plant sources	5.54 ± 0.5	5.50 ± 0.4	0.001
Age at pubertal onset (G2 or B2), years	8.7 ± 1.6	10.0 ± 1.6	< 0.001
Age at pubertal completion (VB or menarche), years	13.6 ± 1.9	12.7 ± 1.2	< 0.001
Age at PHV, years	13.6 ± 0.9	11.7 ± 0.8	< 0.001
Age at P2, years	11.0 ± 1.7	10.8 ± 1.5	< 0.001
Age at AH, years	13.7 ± 1.7	12.2 ± 1.6	< 0.001
Pubertal duration, years	4.8 ± 2.3	2.7 ± 1.3	< 0.001
Body fat percentage, %	21.0 ± 7.8	27.3 ± 7.4	< 0.001

*AH* armpit hair growth, *B2* Tanner stage 2 breast development, *G2* Tanner stage 2 genital development, *PH2* Tanner stage 2 pubic hair growth, *PHV* peak height velocity, *VB* voice breaking

### Dietary associations with puberty timing

Table 2 shows the associations of childhood total energy and macronutrient intakes with subsequent puberty timing in boys and girls, with adjustment for maternal and infant characteristics. In boys, higher TEI was associated with earlier VB (β = − 0.29 years per 500 kcal, 95% Confidence Intervals (CI) = − 0.50, − 0.07). In girls, higher TEI was associated with earlier B2 (β = − 0.28, 95% CI = − 0.46, − 0.10), PHV (β = − 0.15, 95% CI = − 0.26, − 0.04), and menarche (β = − 0.14, 95% CI = − 0.27, − 0.01). Regarding secondary outcomes, higher TEI was associated with earlier PH2 in both sexes (boys: β = − 0.26, 95% CI = − 0.47, − 0.05; girls: β = − 0.19, 95% CI = − 0.38, − 0.01) (Supplementary Table 4).

**Table 2:** Adjusted associations of total energy and macronutrient intakes predicted at age 6 years with puberty timing^a^

Macronutrients	Age at genital/ breast development		Age at peak height velocity		Age at voice breaking/ menarche	
	Adjusted β (95% CI)	*P*	Adjusted β (95% CI)	*P*	Adjusted β (95% CI)	*P*
Boys	*n* = 2619		*n* = 2215		*n* = 3017	
Total energy intake (per 500 kcal increase)	− 0.17 (− 0.36, 0.02)	0.085	− 0.10 (− 0.22, 0.03)	0.129	− 0.29 (− 0.50, − 0.07)	0.009
Energy partition model (per 500 kcal increase)						
Carbohydrates	− 0.42 (− 1.02, 0.18)	0.173	− 0.01 (− 0.37, 0.36)	0.981	− 0.40 (− 1.05, 0.25)	0.231
Fat	0.32 (− 0.47, 1.10)	0.428	− 0.03 (− 0.49, 0.44)	0.910	0.30 (− 0.56, 1.16)	0.490
Protein	− 0.74 (− 2.80, 1.32)	0.479	− 0.69 (− 1.89, 0.52)	0.265	− 1.73 (− 3.96, 0.49)	0.127
Nutrient density model–protein (per 10% increase)^b^	− 0.09 (− 0.75, 0.56)	0.781	− 0.22 (− 0.60, 0.16)	0.261	− 0.29 (− 0.99, 0.41)	0.418
Residual model–protein (per 50 g increase)^c^	− 0.12 (− 1.07, 0.83)	0.809	− 0.27 (− 0.82, 0.28)	0.342	− 0.47 (− 1.49, 0.54)	0.363
Girls	*n* = 3204		*n* = 2509		*n* = 3414	
Total energy intake (per 500 kcal increase)	− 0.28 (− 0.46, − 0.10)	0.003	− 0.15 (− 0.26, − 0.04)	0.008	− 0.14 (− 0.27, − 0.01)	0.040
Energy partition model (per 500 kcal increase)						
Carbohydrates	0.17 (− 0.38, 0.73)	0.543	− 0.21 (− 0.53, 0.12)	0.208	− 0.05 (− 0.45, 0.36)	0.820
Fat	− 0.19 (− 0.94, 0.57)	0.627	0.39 (− 0.05, 0.82)	0.082	0.12 (− 0.42, 0.65)	0.673
Protein	− 2.52 (− 4.45, − 0.59)	0.010	− 1.69 (− 2.81, − 0.57)	0.003	− 1.40 (− 2.79, − 0.02)	0.047
Nutrient density model–protein (per 10% increase)^b^	− 0.88 (− 1.47, − 0.29)	0.003	− 0.48 (− 0.82, − 0.13)	0.007	− 0.41 (− 0.83, 0.01)	0.058
Residual model–protein (per 50 g increase)^c^	− 1.12 (− 2.01, − 0.24)	0.013	− 0.59 (− 1.11, − 0.08)	0.024	− 0.56 (− 1.19, 0.08)	0.084

^a^Adjusted for maternal characteristics (i.e., age at delivery, passive and active smoking during pregnancy, age at menarche, education, pre-pregnancy body mass index, parity), household highest socioeconomic group, and infant characteristics (i.e., birth weight, gestational age, and breastfeeding duration)

^b^Additionally adjusted for energy from fat (%), total energy intake (kcal)

^c^Additionally adjusted for total fat intake (g), total energy intake (kcal)

In energy partition models, higher protein intake was associated with earlier B2 (β = − 2.52 years per 500 kcal, 95% CI = − 4.45, − 0.59), PHV (β = − 1.69, 95% CI = − 2.81, − 0.57) and menarche (β = − 1.40, 95% CI = − 2.79, − 0.02) in girls (Table 2). Higher protein intake was also associated with earlier timing of secondary puberty traits in boys (PH2: β = − 2.94, 95% CI = − 5.07, − 0.80) and girls (PH2: β = − 2.49, 95% CI = − 4.43, − 0.56; AH: β = − 2.75, 95% CI = − 4.63, − 0.86) (Supplementary Table 4). Total carbohydrate and fat intakes were not associated with puberty timing traits, except for higher total fat intake and later AH in girls (β = 1.15, 95% CI = 0.41, 1.89) (Table 2 and Supplementary Table 4). Therefore, for subsequent analyses, we modelled the substitution of protein for carbohydrate intake in puberty timing.

In nutrient density models, the substitution of dietary protein for carbohydrate was associated with earlier B2 (β = − 0.88 years per 10%, 95% CI = − 1.47, − 0.29) and PHV (β = − 0.48, 95% CI = − 0.82, − 0.13), and a tendency to earlier menarche (β = − 0.41, 95% CI = − 0.83, 0.01) in girls, but not with G2, PHV or VB in boys (Table 2). Furthermore, the substitution of dietary protein for carbohydrate was associated with earlier PH2 in both sexes (boys: β = − 0.88, 95% CI = − 1.56, − 0.21; girls: β = − 0.65, 95% CI = − 1.24, − 0.05), and also earlier AH in girls (β = − 0.69, 95% CI = − 1.28, − 0.11) (Supplementary Table 4). These findings were consistent in residual models (Table 2 and Supplementary Table 4).

The above-identified associations were further analysed by age at dietary assessment. Associations between protein intake and puberty timing in girls were consistent across ages (3–7.5 years) (Supplementary Table 5). By contrast, associations between TEI and puberty timing in boy and girls showed moderate heterogeneity by age at dietary assessment, being apparent at 7.5 years old (by 3-day food diary) but not at 3–7 years (by FFQ).

The observed associations between childhood total energy or macronutrient intakes and subsequent puberty timing traits were not attenuated with additional adjustment for body fat percentage at 9–15 years (Supplementary Table 6).

### Categorical associations

More than two-thirds of included children (boys: 68.3%; girls: 73.9%) had predicted TEI at age 6 years higher than the UK EAR. Compared to girls with TEI below EAR, girls with TEI above EAR tended to have higher risk for earlier than later B2 (OR = 1.27, 95% CI = 1.07, 1.50) and menarche (OR = 1.16, 95% CI = 0.98, 1.36) (Supplementary Table 7). All children had higher total protein intakes than the UK recommendation. For each 10 g or 5% increase in dietary protein substituting for carbohydrate intake, girls had ~ 30% higher risks for earlier than later timing of all puberty traits (Supplementary Table 7).

We also explored potential associations of intakes with categorised puberty outcomes. By categorising children into tertiles for each puberty timing trait, we found largely similar effects of intakes on risk of early (versus average) compared to average (versus late) puberty timing, although confidence intervals were wide (Supplementary Table 8). However, higher TEI was associated with lower risk of early versus average G2 in boys (OR = 0.41) but with higher risk of average versus late G2 (OR = 3.59) (Wald test, *p* = 0.009).

### Protein quality

In energy partition models (Supplementary Table 9), while higher plant protein intake was associated with earlier VB in boys, higher intakes of animal protein and plant protein were associated with earlier B2, PHV, and menarche in girls. These associations persisted in nutrient density and residual models (Supplementary Table 9), which tested the substitution of protein types for carbohydrates.

## Discussion

In this long follow-up British study from 3 to 17 years old, we observed that higher childhood habitual intakes of total energy, and of protein at the expense of carbohydrate, were associated with subsequent earlier timings of puberty onset and completion, particularly in girls. Associations between higher TEI and protein intakes with PHV, an objectively measured indicator of puberty timing, were also observed in girls, but not in boys. The findings for protein intake were consistent in different energy adjustment models (energy partition, nutrient density and residual methods). Also, all observed associations persisted after additional adjustment for adiposity during adolescence.

Our findings with TEI and earlier puberty timing are broadly consistent with the previous analysis of ALSPAC which tested only dichotomous age at menarche (before or after 12 years 8 months) and found an association with TEI only at age 10 years (by diet diary) but not at 3 or 7 years (by FFQ) [13]. Similar to that analysis [13], all other reported studies assessed diets at relatively late ages, when many (if not most) girls were already in puberty. A Canadian study (*n* = 666) administered 3-day food diaries at age 10.7 years and recorded menarche status at 11.4 years, showing an inverse association between TEI and age at menarche [12]. Conversely, no association between TEI and categorical or continuous age at menarche was reported by other prospective studies in Canada (3-day food diaries at median age 11.1 years; 17-month follow-up; *n* = 2299 [17]) and The Netherlands (7-day food diaries in 63 pubertal girls, mean age 9.6 years) [19]. A surprising positive association between TEI and continuous age at menarche was reported by a study in Southern California (FFQ at mean age 10.6 years; 4-year follow-up; *n* = 679) [14]. We further added that the inverse association between TEI and puberty timing existed not only in girls but also in boys, and these associations were independent of adiposity during puberty. The present observations suggest that higher TEI may fuel the secretion and bioactivities of reproductive hormones, possibly through effects of leptin and insulin, which in turn accelerates pubertal development [41].

Our findings with dietary protein are broadly consistent with the conclusions of an earlier systematic review in 2013 which reported ‘probable evidence’ for an association between animal protein intakes and earlier puberty in boys and girls [42] based on three prospective studies in the USA [43], UK [13], and Germany [22, 44]. Berkey et al. analysed data on 67 girls in Boston USA and identified that higher protein intakes and specifically animal protein (by dietary history interviews 6 monthly from birth to 11 years) were associated with earlier menarche and PHV [43]. Second, the DONALD study in Germany reported that higher animal protein intake (by 3-day food diaries) at 5–6 years but not earlier 3 time points (*n* = 112), or 1–2 year prior to puberty onset (*n* = 109), was associated with earlier menarche/voice breaking and PHV, without sex stratification [22, 44]. Third, the earlier analysis of the current ALSPAC study reported that higher total and animal protein intakes at 3 and 7 years were associated with higher risk of early menarche [13]. However, in contrast to two of the previous studies which reported opposite associations between higher plant protein intakes and later puberty timing [22, 43], we found similar associations between animal and plant protein intakes and earlier puberty in girls. Unlike previous studies, we performed models that considered all macronutrients together, so identified associations with protein intakes are independent of compensatory changes in the other macronutrients. Furthermore, compared to the earlier ALSPAC report [13], our models included more covariates (maternal age at delivery, passive smoking during pregnancy, pre-pregnancy body mass index, and gestational age). It is difficult to assess whether our approach might have led to over-adjustment, but our findings for TEI and total protein intake remained robust despite the same adjustment, and understanding the role of protein specificity on puberty timing will ultimately rely on experimental studies. Since that review [42], a recent Australian study [20] reported that lower protein intake at 8 years (by 3-day food diaries) was surprisingly associated with earlier puberty in girls (breast development and menarche), which was seen only using nutritional geometry analysis that considers multiple nutrient interactions, but not in linear regression models; however, their sample size was modest (50 boys; 92 girls). Other earlier prospective studies, which reported no association between any macronutrient intake and age at menarche, were not included in the systematic review [42], possibly because they assessed diets at relatively late ages (mean 10.6 [14] and 11.1 years [17]) or in already pubertal girls [19].

The exact mechanisms elucidating the association between protein intake and puberty timing remain to be determined. Our findings of differential associations between boys and girls suggest that there may be sexual dimorphism in protein metabolism [45] underlying pubertal development. Furthermore, the consistent lack of attenuation of associations with additional adjustment for adiposity may suggest direct effects of protein on pubertal onset and/or progression. Higher protein intakes may supply more readily available amino acids, consequently stimulating the production of enzymes and hormones involved in pubertal development including the growth hormone/insulin-like growth factor-1 pathway [46]. Furthermore, high consumption of protein can lead to hyperinsulinemia and insulin resistance [47], which increases bioavailability of sex hormones [9] probably particularly in girls. Notably, plant proteins contributed substantially (~ 40%) to total protein intakes in this cohort, and in most intervention studies, no difference was observed in glucose homeostasis and fasting insulin between plant- and animal-based proteins [48]. Hence, total protein may be as important as protein quality in promoting pubertal development.

We extended existing studies, particularly previous analysis in the same cohort [13], by combining repeated measures of macronutrient intakes during prepubertal childhood (at 3, 4, 7, and 7.5 years) from two assessments of current dietary intakes (i.e., FFQ and 3-day food diaries) and by testing more complete data on various pubertal traits into young adulthood. It is noteworthy that FFQ typically assesses long-term intakes with lower precision, while 3-day food diaries offers higher validity in reference to 9-day food diaries, but is subjected to within-person variations of day-to-day dietary intake and between-person variations of seasonal-to-seasonal dietary intake; neither is considered superior unless validated against reliable biomarkers [49]. We therefore intended to improve accuracy of estimating individual usual intakes during child growth and avoid differential findings by dietary assessments and ages, with consideration of the expected age-related increase in intakes and individual overestimation (by FFQ) and underestimation (by 3-day food diaries) of dietary assessments, using random intercepts linear regressions [32]. Our secondary analyses showed no heterogeneity by specific ages of protein intakes, suggesting a critical window of effect is unlikely. For TEI, the stronger associations at 7.5 years than at 3–7 years are likely due to higher validity of TEI estimation by 3-day food diary than by FFQ. We highlight other differences in our study design and analytical approach compared to previous studies. Many previous studies, including the earlier ALSPAC analysis, examined macronutrient intakes separately, assessed macronutrient intakes at later age when puberty had probably started, considered girls who had not yet completed puberty, and/or used dichotomous menarche status [13–24]. By contrast, we analysed isocaloric substitution of macronutrients throughout prepubertal childhood and estimated continuous timings for a number of puberty traits in both sexes, including PHV as an objective measure of puberty timing. Also, we used multiple imputation to predict missing data mainly in covariates to facilitate the comparisons of findings between models, whereas the previous findings may be subjected to the complete case analyses in adjustment models. Although we predicted macronutrient intakes at age 6 years, our findings represent effects of intakes at ages 3–7.5 years, when the intakes were measured. Similarly, predicted adiposity at 11 years is generalizable to the age range 9–15 years.

Several limitations need to be acknowledged. There were some differences in maternal and infant characteristics between children with and without data on any puberty timing, but the differences were modest and these factors were adjusted for in all models. We could exclude individuals on the basis of implausible values for energy intake only by visual inspection of histograms, because body weight measurements were unavailable in the whole cohort at 3, 4, and 7 years but available only at 7.5 years [50]. However, we included sensitivity analyses (i.e., residual method) to account for potential underreporting of dietary intakes. Furthermore, we synthesised dietary data from several timepoints to reduce random error, using random intercepts linear regression models, rather than a simpler alternative calculation of mean values across visits which could be limited by data which are unbalanced and missing not at random [32]. We acknowledge relatively weak correlations between intakes by FFQ and 3-day food diaries in the present study, as observed elsewhere [49], possibly partly due to differences in ages of assessment. However, our predicted intakes at age 6 years were moderately-to-highly correlated with all reported intakes, and may be considered to provide a balance for the potential overreporting and underreporting of dietary intakes from FFQ and 3-day food diaries, respectively. We found frequent inconsistencies in the repeated self-reports of pubertal development and, even after extensive data cleaning, we were unable to calculate timings of several puberty timing traits in 20–30% of children who otherwise met the inclusion criteria for this analysis. We also note the unusually early average timing of G2 (leading to an unusually long duration of puberty) in boys, and those data should be considered with caution. Nonetheless, our findings are consolidated with the inclusion of PHV as an objective indicator of overall puberty timing. While the present findings highlight TEI, total protein and protein quality, we do not exclude the possibility of the role of types of other macronutrients (i.e., carbohydrate and fat) in puberty timing, which deserves further study. Finally, although our findings were adjusted for several potential confounders, confounding may exist due to unmeasured factors such as body mass index at 3–7 years (which was available only from 7.5 years) and physical activity (which was measured only during adolescence).

In conclusion, we found that higher prepubertal childhood TEI and protein intakes were associated with earlier pubertal development, independent of adiposity during adolescence, more markedly in girls than in boys. These findings suggest potential consequences of the current high levels of TEI and protein intake among children across the world [51, 52] as well as potential explanations for the secular trends towards earlier puberty timing [1–4].

## Appendix 1

Previous studies of the same cohort [28, 29] found that the rates of plausible reporters for 3-day food diaries at age 3–7.5 years ranged from 69 to 76% in the 10% subcohorts (*n* = 772–863) based on the 95% CI [53] of the ratio of reported energy intake to estimated energy requirement (EI:EER) [54]. Similarly, for 3-day food diaries at age 7.5 years (*n* = 7017), we observed only 74% reported plausible energy intake values within the same ratio of EI:EER (0.79–1.21). We also found 79% plausible reporters according to the calculated individual cut-off points [55] of basal metabolic rate estimated using sex-, age-, and body weight-specific Schofield equations [56]. Body weight measures at 3–7 years in the whole cohort were not available.

## Appendix 2

Calculation of puberty timing traits (G2, B2, PH2, AH, VB).

*Ages at onset of genital (G2), breast (B2), pubic hair (PH2), axillary hair (AH), and voice breaking (VB) were calculated from repeated annual self-reports between ages 8 to 17 years.*

Ideally, for each puberty trait, age at onset was calculated as the midpoint age between the first pubertal appearance and the previous annual report (stating prepubertal status):

age at onset = [age at first reported puberty *minus* age at previous annual report]/2

If puberty was already present on the first report at age 8 years:

age at onset = age at 8 years report *minus* 6 months

If puberty was first reported after age 8 years:

age at onset = age at 8 years report *minus* 6 months*

*[the population average interval between the first report and the previous annual report]/2

If puberty was first reported after age 8 years but the one previous annual report was missing:

age at onset = age at first reported puberty *minus* 12 months**

**[the population average interval between the first report and the previous annual report]

If puberty was first reported after age 8 years but the two previous annual reports were missing:

age at onset = age at first reported puberty minus 18 months***

***[the population average interval between the first report and the previous annual report] + 0.5x the midpoint interval between average ages at each annual report for each additional missing report

If puberty was never reported (for VB and axillary hair):

If puberty was not reported as attained at the last report at age 17 years: age at onset = age at 17 years report plus 6 months

If puberty was not reported as attained and subsequent annual reports were missing: the aforementioned time intervals were added to the age at the last reported prepubertal status.

We calculated estimates of puberty timing (G2, B2, PH2, AH, and VB) in individuals whose reported measures followed a consistent sequential order with increasing age (52.2%–98.7% of all included individuals).

Age at VB was calculated in a further 11.8% of boys whose reported measures followed a consistent sequential order for at least three consecutive visits before or after an inconsistent report (e.g., ‘no VB’ occurring after ‘partial or total VB’).

For each of G2, B2, VB, and PH2, timing of puberty could not be confidently calculated in 20.7%–31.7% of individuals, due to inconsistent reports (i.e., non-sequential ordering of pubertal status and/or missing data).

## Data Availability

All ALSPAC data are available to scientists on request to the ALSPAC Executive via this website, which also provides full details and distributions of the ALSPAC study variables: http://www.bristol.ac.uk/alspac/researchers/access/.

## References

[1] Hosokawa M, Imazeki S, Mizunuma H, Kubota T, Hayashi K (2012). Secular trends in age at menarche and time to establish regular menstrual cycling in Japanese women born between 1930 and 1985. BMC Womens Health.

[2] Herman-Giddens ME, Slora EJ, Wasserman RC, Bourdony CJ, Bhapkar MV, Koch GG, Hasemeier CM (1997). Secondary sexual characteristics and menses in young girls seen in office practice: a study from the Pediatric Research in Office Settings network. Pediatrics.

[3] Herman-Giddens ME, Steffes J, Harris D, Slora E, Hussey M, Dowshen SA, Wasserman R, Serwint JR, Smitherman L, Reiter EO (2012). Secondary sexual characteristics in boys: data from the Pediatric Research in Office Settings Network. Pediatrics.

[4] Brix N, Ernst A, Lauridsen LLB, Parner E, Støvring H, Olsen J, Henriksen TB, Ramlau-Hansen CH (2019). Timing of puberty in boys and girls: a population-based study. Paediatr Perinat Epidemiol.

[5] Galvao TF, Silva MT, Zimmermann IR, Souza KM, Martins SS, Pereira MG (2014). Pubertal timing in girls and depression: a systematic review. J Affect Disord.

[6] Day FR, Elks CE, Murray A, Ong KK, Perry JR (2015). Puberty timing associated with diabetes, cardiovascular disease and also diverse health outcomes in men and women: the UK Biobank study. Sci Rep.

[7] Prentice P, Viner RM (2013). Pubertal timing and adult obesity and cardiometabolic risk in women and men: a systematic review and meta-analysis. Int J Obes (Lond).

[8] Cheng TS, Day FR, Lakshman R, Ong KK (2020). Association of puberty timing with type 2 diabetes: A systematic review and meta-analysis. PLoS Med.

[9] Ahmed ML, Ong KK, Dunger DB (2009). Childhood obesity and the timing of puberty. Trends Endocrinol Metab.

[10] Fan HY, Huang YT, Hsieh RH, Chao JC, Tung YC, Lee YL, Chen YC (2018). Birthweight, time-varying adiposity growth and early menarche in girls: a Mendelian randomisation and mediation analysis. Obes Res Clin Pract.

[11] Villamor E, Jansen EC (2016). Nutritional determinants of the timing of puberty. Annu Rev Public Health.

[12] Moisan J, Meyer F, Gingras S (1990). A nested case-control study of the correlates of early menarche. Am J Epidemiol.

[13] Rogers IS, Northstone K, Dunger DB, Cooper AR, Ness AR, Emmett PM (2010). Diet throughout childhood and age at menarche in a contemporary cohort of British girls. Public Health Nutr.

[14] Koprowski C, Ross RK, Mack WJ, Henderson BE, Bernstein L (1999). Diet, body size and menarche in a multiethnic cohort. Br J Cancer.

[15] Petridou E, Syrigou E, Toupadaki N, Zavitsanos X, Willett W, Trichopoulos D (1996). Determinants of age at menarche as early life predictors of breast cancer risk. Int J Cancer.

[16] Meulenijzer E, Vyncke K, Labayen I (2015). Associations of early life and sociodemographic factors with menarcheal age in European adolescents. Eur J Pediatr.

[17] Moisan J, Meyer F, Gingras S (1990). Diet and age at menarche. Cancer Causes Control.

[18] Meyer F, Moisan J, Marcoux D, Bouchard C (1990) Dietary and physical determinants of menarche. Epidemiology 377–381 10.1097/00001648-199009000-00007 2078613

[19] de Ridder CM, Thijssen JH, Van’t Veer P, van Duuren R, Bruning PF, Zonderland ML, Erich WB (1991). Dietary habits, sexual maturation, and plasma hormones in pubertal girls: a longitudinal study. Am J Clin Nutr.

[20] Cheng HL, Raubenheimer D, Steinbeck K, Baur L, Garnett S (2019). New insights into the association of mid-childhood macronutrient intake to pubertal development in adolescence using nutritional geometry. Br J Nutr.

[21] Kissinger D, Sanchez A (1987). The association of dietary factors with the age of menarche. Nutr Res.

[22] Gunther AL, Karaolis-Danckert N, Kroke A, Remer T, Buyken AE (2010). Dietary protein intake throughout childhood is associated with the timing of puberty. J Nutr.

[23] Merzenich H, Boeing H, Wahrendorf J (1993). Dietary fat and sports activity as determinants for age at menarche. Am J Epidemiol.

[24] Koo MM, Rohan TE, Jain M, McLaughlin JR, Corey PN (2002). A cohort study of dietary fibre intake and menarche. Public Health Nutr.

[25] Berenbaum SA, Beltz AM, Corley R (2015). The importance of puberty for adolescent development: conceptualization and measurement. Adv Child Dev Behav.

[26] Boyd A, Golding J, Macleod J, Lawlor DA, Fraser A, Henderson J, Molloy L, Ness A, Ring S, Davey Smith G (2013). Cohort profile: the 'children of the 90s'–the index offspring of the avon longitudinal study of parents and children. Int J Epidemiol.

[27] Fraser A, Macdonald-Wallis C, Tilling K, Boyd A, Golding J, Davey Smith G, Henderson J, Macleod J, Molloy L, Ness A, Ring S, Nelson SM, Lawlor DA (2013). Cohort profile: the avon longitudinal study of parents and children: ALSPAC mothers cohort. Int J Epidemiol.

[28] Emmett PM, Jones LR, Northstone K (2015). Dietary patterns in the avon longitudinal study of parents and children. Nutri Rev.

[29] Emmett PM, Jones LR (2015). Diet, growth, and obesity development throughout childhood in the avon longitudinal study of parents and children. Nutri Rev.

[30] Welch AA, Luben R, Khaw K, Bingham S (2005). The CAFE computer program for nutritional analysis of the EPIC-Norfolk food frequency questionnaire and identification of extreme nutrient values. J Hum Nutr Diet.

[31] Price G, Paul A, Key F, Harter A, Cole T, Day K, Wadsworth M (1995). Measurement of diet in a large national survey: comparison of computerized and manual coding of records in household measures. J Hum Nutr Diet.

[32] Chen YH, Ferguson KK, Meeker JD, McElrath TF, Mukherjee B (2015). Statistical methods for modeling repeated measures of maternal environmental exposure biomarkers during pregnancy in association with preterm birth. Environ Health.

[33] Petersen AC, Crockett L, Richards M, Boxer A (1988). A self-report measure of pubertal status: Reliability, validity, and initial norms. J Youth Adolesc.

[34] Frysz M, Howe LD, Tobias JH, Paternoster L (2018). Using SITAR (SuperImposition by Translation and Rotation) to estimate age at peak height velocity in avon longitudinal study of parents and children. Wellcome Open Res.

[35] Ong KK (2017). What triggers puberty?. Arch Dis Child.

[36] Willett WC, Howe GR, Kushi LH (1997). Adjustment for total energy intake in epidemiologic studies. Am J Clin Nutr.

[37] Voss S, Kroke A, Klipstein-Grobusch K, Boeing H (1998). Is macronutrient composition of dietary intake data affected by underreporting? Results from the EPIC-Potsdam Study. European Prospective Investigation into Cancer and Nutrition. Eur J Clin Nutr.

[38] Scientific Advisory Committee on Nutrition (2011) Dietary reference values for energy

[39] Public Health England (2016). Government recommendations for energy and nutrients for males and females aged 1–18 years and 19+ years. https://assets.publishing.service.gov.uk/government/uploads/system/uploads/attachment_data/file/618167/government_dietary_recommendations.pdf

[40] Graham JW, Olchowski AE, Gilreath TD (2007). How many imputations are really needed? Some practical clarifications of multiple imputation theory. Prev Sci.

[41] Garcia-Garcia RM (2012). Integrative control of energy balance and reproduction in females. ISRN Vet Sci.

[42] Hörnell A, Lagström H, Lande B, Thorsdottir I (2013). Protein intake from 0 to 18 years of age and its relation to health: a systematic literature review for the 5th Nordic Nutrition Recommendations. Food Nutr Res.

[43] Berkey CS, Gardner JD, Frazier AL, Colditz GA (2000). Relation of childhood diet and body size to menarche and adolescent growth in girls. Am J Epidemiol.

[44] Remer T, Shi L, Buyken AE, Maser-Gluth C, Hartmann MF, Wudy SA (2010). Prepubertal adrenarchal androgens and animal protein intake independently and differentially influence pubertal timing. J Clin Endocrinol Metab.

[45] Markofski MM, Volpi E (2011). Protein metabolism in women and men: similarities and disparities. Curr Opin Clin Nutr Metab Care.

[46] Brosnan ME, Brosnan JT, Young VR (2011). Integration of Metabolism 3: Protein and Amino Acids. Nutr Metab.

[47] Rietman A, Schwarz J, Tomé D, Kok FJ, Mensink M (2014). High dietary protein intake, reducing or eliciting insulin resistance?. Eur J Clin Nutr.

[48] Chalvon-Demersay T, Azzout-Marniche D, Arfsten J, Egli L, Gaudichon C, Karagounis LG, Tomé D (2017). A systematic review of the effects of plant compared with animal protein sources on features of metabolic syndrome. J Nutr.

[49] Yang YJ, Kim MK, Hwang SH, Ahn Y, Shim JE, Kim DH (2010). Relative validities of 3-day food records and the food frequency questionnaire. Nutr Res Pract.

[50] Vainik U, Konstabel K, Latt E, Maestu J, Purge P, Jurimae J (2016). Diet misreporting can be corrected: confirmation of the association between energy intake and fat-free mass in adolescents. Br J Nutr.

[51] Suthutvoravut U, Abiodun PO, Chomtho S (2015). composition of follow-up formula for young children aged 12–36 months: Recommendations of an International Expert Group Coordinated by the Nutrition Association of Thailand and the Early Nutrition Academy. Ann Nutr Metab.

[52] Bates B, Lennox A, Prentice A, Bates C, Swan G (2014) National Diet and Nutrition Survey: Results from Years 1, 2, 3 and 4 (combined) of the Rolling Programme (2008/2009–2010/11): A survey carried out on behalf of the Department of Health and the Food Standards Agency. Public Health England.

[53] Black AE, Cole TJ (2000). Within- and between-subject variation in energy expenditure measured by the doubly-labelled water technique: implications for validating reported dietary energy intake. Eur J Clin Nutr.

[54] Torun B (2005). Energy requirements of children and adolescents. Public Health Nutr.

[55] Torun B (1996). Energy requirements and dietary energy recommendations for children and adolescents 1 to 18 years old. Eur J Clin Nutr.

[56] Schofield WN, Schofield C, James WPT (1985) Basal metabolic rate: review and prediction, together with an annotated bibliography of source material. 3900006

